# Sexual Dysfunction in Postural Orthostatic Tachycardia Syndrome (POTS): A Cross-Sectional, Case-Control Study

**DOI:** 10.3390/jcm13082274

**Published:** 2024-04-14

**Authors:** Svetlana Blitshteyn, Anna Lange, Chelsea Corinaldi, Paige Guy, Jill Brook

**Affiliations:** 1 Department of Neurology, Jacobs School of Medicine and Biomedical Sciences, University at Buffalo, Buffalo, NY 14203, USA; 2 Dysautonomia Clinic, Williamsville, NY 14221, USA

**Keywords:** postural orthostatic tachycardia syndrome (POTS), sexual dysfunction, case–control study

## Abstract

**Background:** We aimed to determine whether patients with postural orthostatic tachycardia syndrome (POTS) have sexual dysfunction compared to age-matched healthy controls. **Methods:** Utilizing online COMPASS-31 to evaluate dysautonomia symptom severity, Beck’s Depression Inventory Second Edition (BDII), Female Sexual Function (FSF), and International Index of Erection Function (IIEF) questionnaires, we compared sexual function scores in patients with POTS to scores obtained from sex- and age-matched healthy controls via a cross-sectional case–control study. **Results:** A total of 160 women with POTS, mean age 30.2 ± 7.9 (range 21–50 years), had lower FSF scores than 62 healthy age-matched female controls. IIEF scores in 29 male patients with POTS with a mean age of 30.1 ± 6.0 (range 21–47) were significantly lower than in 27 healthy age-matched male controls. Female POTS patients had significantly lower scores in the sub-domains of desire, arousal, and satisfaction, while male POTS patients had significantly lower scores in erectile and orgasmic function, desire, and satisfaction than healthy controls. Predictive factors of sexual dysfunction were depression in women and age in men. The severity of autonomic symptoms correlated with sexual dysfunction in women, but this effect disappeared after controlling for depression. **Conclusions:** Compared to healthy controls, women and men with POTS have significant sexual dysfunction, which needs to be considered in the diagnostic and therapeutic approaches as part of comprehensive patient care.

## 1. Introduction

Postural orthostatic tachycardia syndrome (POTS), one of the most common disorders of the autonomic nervous system, is characterized by postural tachycardia with a heart rate increase greater than 30 bpm from supine to standing position and without accompanying orthostatic hypotension. POTS is defined by symptoms of orthostatic intolerance, such as dizziness, lightheadedness, and palpitations, and is often associated with non-orthostatic manifestations, such as fatigue, headaches, gastrointestinal dysfunction, and cognitive impairment (also known as “brain fog”). POTS predominantly affects women of reproductive age and is commonly triggered by growth spurts, menarche, pregnancy, concussion, surgery, vaccination, and infection, including SARS-CoV-2 (1). In conjunction with clinical history, POTS is diagnosed via a 10 min stand test performed at bedside or a tilt table test. Physical exams are often unremarkable but may show dilated pupils, dry skin, reduced pain, and/or temperature sensation in the feet, suggestive of small-fiber neuropathy, or acrocyanosis, which is a purple-bluish discoloration of the feet and hands, especially in the dependent position.

Clinical experience and several studies concerning pregnancy and gynecologic complications suggest that sex hormones play an important role in symptoms and disease course in patients with POTS [1,2,3]. Since patients with POTS tend to be young women and men in the prime of their reproductive life, sexual function represents an important consideration, both from the quality of life and reproductive health standpoints.

Sexual dysfunction is characterized by disturbances in sexual response, desire, or orgasm or pain that causes distress, and it is prevalent in patients with various chronic conditions, including neurologic disorders such as multiple sclerosis, epilepsy, Parkinson’s disease, and chronic migraine [4]. We aimed to assess sexual function in patients with POTS, as well as possible predictive factors associated with sexual dysfunction, and to determine which aspects of sexual dysfunction, if present, are prevalent in women and men with POTS. 

## 2. Methods

### 2.1. Study Participants

Patients with POTS were recruited from social media, online patient support groups, and the Dysautonomia Clinic. Inclusion criteria were a self-reported physician-made diagnosis of POTS, age 18 and older, and being sexually active in the past 4 weeks. Exclusion criteria were autonomic disorders other than POTS, such as neurocardiogenic syncope, orthostatic hypotension, or unspecified autonomic dysfunction, and being sexually inactive or abstinent in the past 4 weeks. Participants were required to have a diagnosis of POTS made either via a tilt table test or a 10 min stand test performed at a doctor’s office. A diagnosis of POTS was made based on the following standard diagnostic criteria: (1) Sustained heart rate increase of ≥30 bpm within 10 min of assuming upright posture. (2) No evidence of orthostatic hypotension on a tilt table test or a 10 min stand test. (3) Symptoms of orthostatic intolerance (OI) present for ≥6 months.

Healthy controls were recruited among medical students at the University of Buffalo Jacobs School of Medicine, through social media, and from referrals of POTS participants, which consisted of healthy individuals unrelated to POTS participants. Only healthy participants without POTS and without major medical conditions, such as hypertension, diabetes, rheumatoid arthritis, and other chronic disorders, were asked to complete the questionnaires.

All study participants consented to participate in the study. The study was approved by the Institutional Review Board at the University at Buffalo Jacobs School of Medicine.

### 2.2. Surveys

All participants completed the Beck Depression Inventory (BDI-II), which contains 21 multiple-choice questions and results in a score between 0 and 63, with higher scores indicating more depressive symptoms [5]. To control for questions asking for somatic symptoms that overlap with symptoms of POTS, we created a second ”POTS-aware” scoring of the BDI-II, which eliminated 5 questions (#15 loss of energy, #16 change in sleep, #18 change in appetite, #19 concentration difficulty, #20 fatigue), for a possible total score of 0–48 points [6].

Self-identified women completed the Female Sexual Function Index (FSFI), which consists of 19 multiple-choice questions and renders a total score between 2 and 36 points, with higher scores indicating better sexual function. Subscores, with up to 6 points each, were calculated for the following domains: desire, arousal, lubrication, orgasm, satisfaction, and pain [7]. 

Self-identified men completed the International Index of Erectile Function (IIEF), which contains 15 multiple-choice questions and renders a total score between 0 and 75. Subscores (maximum scores) are assigned in the domains of erectile function (30), orgasmic function (10), sexual desire (10), intercourse satisfaction (15), and overall satisfaction (10) [8]. 

Patients completed the Composite Autonomic Symptom Score-31 (COMPASS-31) questionnaire, which is a validated tool to evaluate autonomic symptom burden, consisting of 31 multiple-choice questions grouped into six domains: orthostatic intolerance, vasomotor, secretomotor, gastrointestinal, bladder, and pupillomotor dysfunction. Each answer is assigned a score, and scores within a domain are totaled to obtain a raw domain score. Scores are then multiplied by a weighting factor, based on the relevance of each domain for assessing autonomic function, to yield the final domain score. The maximum weighted domain scores are as follows: orthostatic intolerance 40; vasomotor 5; secretomotor 15; gastrointestinal 25; bladder 10; pupillomotor 5, yielding a total score range of 0–100 [9]. 

### 2.3. Additional Cohort

In addition, we conducted a separate sample study by recruiting qualifying patients from the Dysautonomia Clinic, all with a diagnosis of POTS confirmed by one investigator (SB) either via a 10 min stand test or a tilt table test, in order to verify an association between a diagnosis of POTS and sexual dysfunction and to compare results to online patients. The control group included patients’ unrelated healthy friends without POTS or other chronic illnesses and healthy medical students from the University at Buffalo Jacobs School of Medicine. Healthy controls were recruited via invitation by either patients or investigators of the Dysautonomia Clinic. In this separate study, patients and controls completed and returned the questionnaires anonymously via e-mail.

### 2.4. Statistical Analyses

All tests were performed separately for males and females. Welch’s two-sample *t*-tests with Cohen’s D effect size calculation compared POTS patients to controls on mean age, Beck depression scores, global sexual function score, and each of the sub-domain scores. Pearson’s correlations examined the associations between age, depression score, sexual function score, and COMPASS-31 score. Multiple regression models analyzed whether age, COMPASS-31 scores, or BDI-II scores predicted sexual dysfunction in POTS patients. Analyses were performed using the statistical software R (v4.1.2; R Core Team 2021), and statistical significance was defined as *p*  <  0.05. 

## 3. Results

### 3.1. Online Patient Characteristics

The participating 160 female POTS patients had a mean age of 30.2 ± 7.9 (range 21–50 years), which did not significantly differ from that of the 62 female controls (29.1 ± 4.6; range 21–47; *p* = 0.5). The participating 29 male POTS patients had a mean age of 30.1 ± 6.0 (range 21–47), which did not significantly differ from that of the 27 male controls (29.8 ± 5.6; range 21–48; *p* = 0.6). Patient characteristics and mean scores are outlined in Table 1 and Table 2.

Female patients’ COMPASS-31 scores ranged from 22 to 82 with a mean of 55.3 ± 11.8. Male patients’ COMPASS-31 scores ranged from 6 to 63 with a mean of 46.4 ± 6.5. 

### 3.2. Specialty Clinic Characteristics

Dysautonomia Clinic patient characteristics and mean scores are outlined in Table 3. The participating 11 female POTS patients from Dysautonomia Clinic had a mean age of 40.18 ± 9.37 (range 21–50 years), which was not significantly different from the seven controls (mean 36.00 ± 13.8, *p* = 0.45). This cohort of patients’ COMPASS-31 scores ranged from 35 to 62 with a mean of 47.55 ± 13.91.

### 3.3. Depression Scores

POTS patients of both genders had significantly higher depression scores than controls. Female patients had a mean score of 22.9 ± 10.7, versus 8.2 ± 6.8 for controls [t (174) = 12.2, *p* < 0.0001]. Male POTS patients had a mean score of 22.7 ± 9.8, versus 11.0 ± 11.6 for controls [t (51) = 4.1, *p* = 0.0002]. 

Using the POTS-aware scoring, this effect remained. Female patients had a mean score of 15.1 ± 8.6, versus 5.9 ± 5.4 for controls [t (176) = 9.6, *p* < 0.0001]. Male POTS patients had a mean score of 16.2 ± 7.5, versus 7.6 ± 5.9 for controls [t (52) = 4.1, *p* = 0.0001].

Question #21 of the Beck Depression Inventory asked specifically about loss of interest in sex. Female patients reported significantly more loss of interest in sex than controls (1.1 ± 1.0 vs. 0.39 ± 0.7, t (162) = 6.1, *p* < 0.0001), as did male patients (1.2 ± 1.0 vs. 0.6 ± 0.8, t (53) = 2.4, *p* = 0.017). The mean depression score amongst the 11 female POTS patients from the specialty clinic was 15.45 ± 10.15. The mean score amongst the seven controls consisting of medical students was 10.57 ± 9.54.

### 3.4. Female Sexual Function Scores

**Figure 1:**
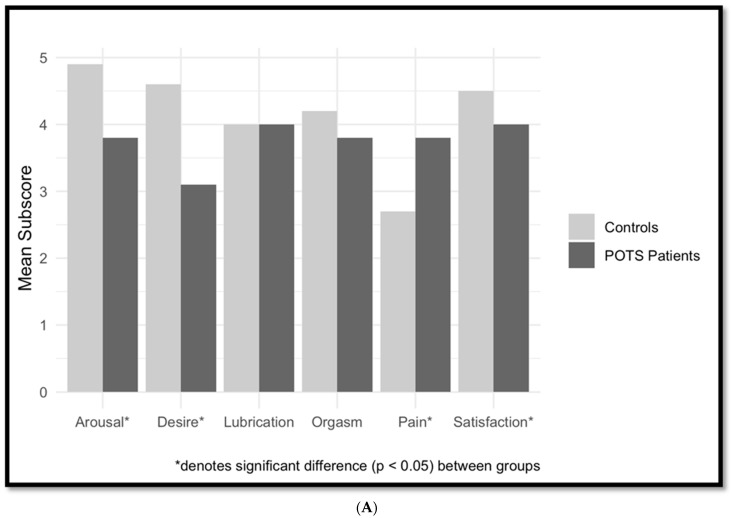

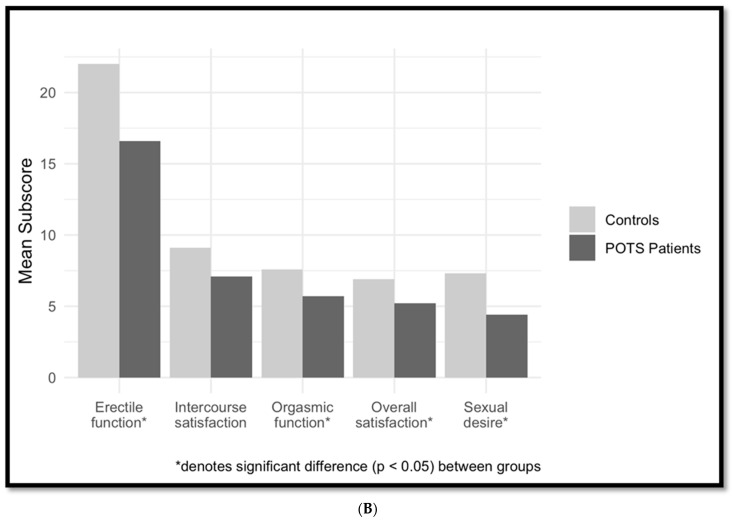
(**A**) Female Sexual Function subscores for female online cohort of POTS patients (N = 160) vs. female controls (N = 62). (**B**) IIEF subscores for male online POTS patients (N = 29) vs. male controls (N = 27).

Female Sexual Function scores were significantly lower in POTS patients than in controls [22.4 ± 6.9 vs. 24.8 ± 5.7, t (135) = −2.7, *p* = 0.0085]. As shown in Figure 1, POTS patients had significantly more sexual dysfunction in the desire, arousal, and satisfaction domains. There were no significant differences in lubrication or orgasm, and POTS patients scored better than controls on pain (3.8 ± 1.8 vs. 2.7 ± 1.9, t (106) = 3.9, *p* = 0.0001).

Among female POTS patients, sexual functioning scores showed a significant negative association with depression scores (r = −0.21, *p* = 0.0082) and COMPASS-31 scores (r = −0.28, *p* = 0.0003), and no significant association with age (r = −0.02, *p* = 0.6240).

Female Sexual Function scores in the Dysautonomia Clinic cohort are outlined in Table 3. Female POTS patients had a mean Female Sexual Function score of 19.47 ± 6.65, significantly lower than healthy controls (mean FSF score of 27.08 ± 5.58 t (16) = 2.5, *p* = 0.02.). 

POTS patients in the Dysautonomia Clinic cohort had significantly worse scores on total FSFI and in the sub-domains of satisfaction and pain. There was a non-significant trend (*p* = 0.1) for lubrication and desire. FSF sub-domain scores are outlined in Table 3 and Figure 1B. Among female POTS patients of the Dysautonomia Clinic, there were no significant correlations between age, BDII, FSFI, or COMPASS-31.

### 3.5. Male Sexual Function Scores

Global sexual function scores were significantly lower in male POTS patients (39.7 ± 11.8) compared to male controls (54.7 ± 24.6; t (44) = −3.7, *p* = 0.0007) (Table 2). The following domains were significantly lower in male POTS patients: erectile function (*p* = 0.0011), orgasmic function (*p* = 0.0170), sexual desire (*p* < 0.0001), and overall satisfaction (*p* = 0.0118). Intercourse satisfaction showed a similar trend toward significance (*p* = 0.0620).

Among male POTS patients, sexual functioning scores showed a significant association with age (r = 0.43, *p* = 0.0186), but not COMPASS-31 scores (r = 0.26, *p* = 0.1679) or BDI-II scores (r = 0.08, *p* = 0.6691) (Table 4).

### 3.6. Predictors of Sexual Dysfunction in POTS 

**Figure 2:**
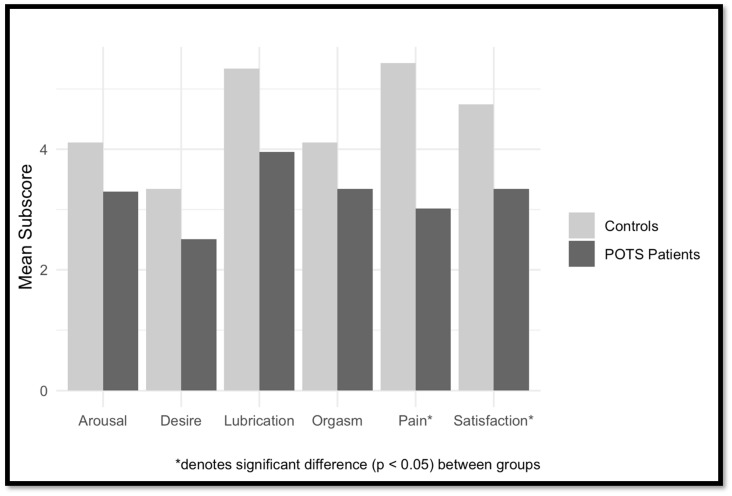
Female Sexual Function subscores for specialty clinic female POTS patients (N = 11) vs. female controls (N = 7).

Among female POTS patients (Figure 2), multiple linear regression analysis between the FSFI score as a dependent variable and COMPASS-31, age, and BDI-II as independent variables showed BDI-II to be an independent predictor of FSFI. Severity of autonomic symptoms correlated with sexual dysfunction in women, but this effect disappeared after controlling for depression. Among male POTS patients, multiple linear regression analysis between the IIEF as a dependent variable and COMPASS-31, age, and BDI-II as independent variables showed age to be an independent predictor of IIEF (Table 5). 

## 4. Discussion

### 4.1. Main Findings

Using validated questionnaires to assess sexual dysfunction, depression, and autonomic symptom severity, we found significant sexual dysfunction in women and men with POTS compared to healthy controls. Female POTS patients had significantly lower scores in the sub-domains of desire, arousal, and satisfaction, while male POTS patients had significantly lower scores in erectile and orgasmic function, desire, and overall satisfaction than healthy controls. To our knowledge, this is the first study that examined sexual function in patients with POTS.

We found a higher rate of depression among female and male patients with POTS compared to controls. Lower Female Sexual Function scores were correlated with higher depression scores and more severe POTS symptoms. Male POTS patients had a significant association between sexual dysfunction and age, but not depression or severity of autonomic dysfunction. Male patients also had lower COMPASS-31 scores than female patients, which parallels our clinical experience of men generally having less severe POTS than women. To our knowledge, this is the largest cohort to date comparing COMPASS-31 scores between women and men with POTS.

Furthermore, comparing scores from a small cohort of Dysautonomia Clinic patients with scores from a large cohort of online participants, clinic patients had significantly lower total COMPASS-31 scores, indicating a lesser burden of autonomic symptoms; significantly lower BDI-II scores, indicating lower levels of depressive symptoms; and lower FSF scores, indicating greater sexual dysfunction compared to online participants. While a larger clinic cohort would have been preferable, these preliminary findings suggest that similar to online participants, female patients with POTS seen at a specialty clinic also have significant sexual dysfunction, which, unlike in online participants, does not appear to be directly related to depressive symptoms. Additionally, patients with POTS from a specialty clinic had lower autonomic and depressive symptoms compared to online community patients, possibly suggesting better disease control via specialized POTS care. 

Finally, pain scores were significantly higher in patients with POTS than controls from the Dysautonomia Clinic, but not among the online participants with POTS. This discrepancy could be due, in part, to a higher prevalence of comorbid Ehlers–Danlos syndrome and small-fiber neuropathy—both of which are associated with dyspareunia, vulvodynia, and other pelvic pain conditions—in Dysautonomia Clinic patients than among online participants. Surprisingly, pain scores were actually lower in online participants with POTS than in online healthy controls, an unexpected finding that deserves further exploration. Nevertheless, similar to online participants, sexual dysfunction among patients from a specialty clinic remained significant, necessitating the development of targeted diagnostic and therapeutic approaches to address this highly prevalent unmet need.

In summary, the study findings are highly relevant because sexual dysfunction can significantly impact reproduction, quality of life, intimate relationships, and overall well-being. Identifying sexual dysfunction in patients with POTS is essential to providing multidisciplinary comprehensive patient care and to elucidating the multifaceted nature of the disorder beyond postural tachycardia. 

### 4.2. Sexual Function and Autonomic Nervous System

Sexual functioning is intricately linked to the autonomic nervous system, which regulates sexual arousal and response. Sympathetic and parasympathetic fibers travel via the pelvic and pudendal nerves to innervate the pelvic organs. The balance between sympathetic and parasympathetic supply is crucial for generating phases of sexual response, such as desire, arousal, plateau, orgasm, and resolution [10,11]. During sexual arousal, the sympathetic nervous system triggers the release of hormones such as norepinephrine, epinephrine, and dopamine to increase heart rate and blood pressure and promote the relaxation of smooth muscles in the genital and pelvic regions. The parasympathetic nervous system stimulates the release of nitric oxide, which relaxes blood vessels in the genitalia, leading to increased blood flow to the penis or clitoris, and resulting in erection or engorgement. The autonomic nervous system also interacts with emotional and psychological factors to influence sexual desire and overall sexual well-being [10,11]. Various hormones and neurotransmitters are involved in regulating sexual function as well. Estrogen is crucial for sexual functioning in women, playing a role in lubrication and sexual desire. Testosterone is the primary driver of sexual functioning in men, although estrogen also plays a role in modulating sexual desire and erectile function. Oxytocin is released in large amounts during sexual activity in both men and women, promoting bonding between partners, relaxation, and sexual receptivity. Other neurotransmitters such as serotonin can affect sexual function. Given the complexity of normal sexual functioning, there are many opportunities for disruption. For example, erectile dysfunction can be due to neurological impairment of the parasympathetic nervous system, vascular problems preventing adequate blood flow, endothelial dysfunction, decreased testosterone levels, or psychological factors [12]. Similarly, premature ejaculation can occur from overactivity of the sympathetic nervous system, neurotransmitter imbalances, or anxiety [13].

### 4.3. Sexual Dysfunction and Neurologic Disorders

Sexual dysfunction is prevalent among individuals with various chronic diseases, including multiple sclerosis, chronic migraine, and multiple system atrophy (MSA)—a progressive, neurodegenerative autonomic disorder with a poor prognosis. A study by Raccagni et al. found significant sexual dysfunction in women with MSA, particularly affecting desire, arousal, and lubrication. These findings parallel the sexual dysfunction seen in men with MSA, which is part of the urogenital dysfunction diagnostic criteria [14]. Sexual dysfunction was seen in up to two-thirds of women with multiple sclerosis and migraine, with depression being a predictive factor for the severity of sexual dysfunction, similar to findings in our study [15]. 

Women with POTS experience increased lightheadedness throughout all phases of the menstrual cycle, with the most significant lightheadedness during menses [3]. There is a higher incidence of gynecologic disorders found among patients with POTS, suggesting a potential link between estrogen fluctuations and POTS symptoms [3]. Moreover, the literature on POTS and pregnancy reveals that pregnancy can precipitate POTS in at least 9% of patients or exacerbate pre-existing POTS in at least half of pregnant women [1,2]. These observations demonstrate the significant role hormonal changes may play in autonomic dysfunction observed in POTS, which can similarly contribute to sexual dysfunction.

Additionally, common comorbidities of POTS, such as Ehlers–Danlos syndrome, migraine, and autoimmune disorders, also appear to be associated with gynecologic abnormalities and/or sexual dysfunction. Specifically, a high incidence of dyspareunia and vulvodynia was found in women with EDS and other hypermobility syndromes, with as many as half experiencing vulvodynia, compared to about 8% in the general US population [16]. Given a high prevalence of comorbidity between POTS and EDS, there may also be increased rates of these gynecologic complications in POTS patients. Additionally, a systematic review revealed that 63% of women with systemic autoimmune rheumatic diseases, another common comorbidity in POTS, experience sexual dysfunction, which is particularly prevalent in patients with Sjogren’s syndrome and systemic sclerosis [17].

POTS and other forms of autonomic dysfunction commonly follow SARS-CoV-2 infection and are a major component of post-acute sequelae of SARS-CoV-2 (PASC), also known as Long COVID [18,19]. One study found that the rate of sexual dysfunction was 50.3% at three months post-recovery in men who had been hospitalized with COVID-19, with major depression being significantly associated with erectile dysfunction for men over 40 years of age [20]. Another study found ejaculation difficulty and reduced libido to be among the top symptoms significantly associated with SARS-CoV-2 infection at 12 weeks in people with Long COVID [21].

Migraine is one of the most common comorbidities in POTS, affecting at least 40% of patients [22], and it has been investigated in relation to sexual function more thoroughly than other disorders. Several studies examined the prevalence and characteristics of sexual dysfunction in migraine patients [23,24,25]. Similar to our findings, these studies found that women with migraine had sexual dysfunction and lower levels of sexual satisfaction. Specifically, women reported difficulties with sexual desire, arousal, pain, and orgasm [23,25]. The relationship between migraine, dysautonomia, and sexual dysfunction may involve shared underlying biological mechanisms, including autonomic dysregulation in all three disorders [26]. Similar to our study, Eraslan et al. found that the most important factor that predicted sexual function in women with migraine was depression, which was independent of disease severity and migraine-related disability [27]. In a study of male patients, IIEF scores of men with migraine and men with tension-type headaches were significantly lower compared to the healthy control group, but no direct relation of erectile dysfunction with Beck depression scores and body mass index was found in male headache patients [28].

In summary, based on our study, sexual dysfunction appears to be prevalent and significant in patients with POTS, as in patients with migraine, which likely adversely affects quality of life, relationship satisfaction, and possibly reproductive functions. Sex hormones play an important role in the pathophysiology and disease course of both POTS and migraine and may be involved in reproductive and sexual functions in women and men affected by both disorders. Further research is needed to examine the hormonal profile in patients with POTS, to understand the complex relationship between autonomic dysregulation and sexual dysfunction, and to identify effective interventions for improving sexual health outcomes. Longitudinal studies examining changes in sexual function over time in response to POTS treatment and exploring the mechanisms underlying sexual dysfunction in patients with autonomic disorders in general are warranted. Understanding the multifaceted relationship between dysautonomia and sexual function is essential for developing targeted interventions to address this unrecognized and unmet need in the management of patients with POTS.

### 4.4. Strengths and Limitations

This cross-sectional study with a large sample size included validated questionnaires and a control group as well as a separate cohort of patients with a verified POTS diagnosis to ensure the accuracy of the association between POTS and sexual dysfunction. 

Limitations of this study include the self-reported nature of the questionnaire responses and inclusion criteria that required being sexually active in the past 4 weeks, thus decreasing generalizability and introducing selection bias by excluding potentially sicker patients who are not sexually active due to severe symptoms and disability. To ensure that the association between sexual dysfunction and POTS was accurate, we verified findings obtained from online responders by assessing a small patient cohort from the Dysautonomia Clinic and comparing them to healthy age-matched controls; the results were similar between unverified online responders self-reporting POTS and Dysautonomia Clinic patients with a verified POTS diagnosis, with sexual dysfunction found in both cohorts. With respect to the limiting inclusion criteria, further studies are needed to determine the prevalence of patients who are sexually inactive and the reasons underlying their sexual abstinence. Finally, in this study, we did not account for a possible effect of POTS comorbidities or medications taken by the study participants, such as beta blockers and antidepressants, which can have various adverse sexual side effects and can also result in sexual dysfunction. These factors and their effects are best examined in the context of prospective cohort studies where sexual function can be compared between patients on and off medications.

Despite these limitations, our study is the first to investigate sexual function in patients with POTS and to demonstrate significant sexual dysfunction in women and men with POTS compared to healthy controls. These findings reveal an unmet need, which calls for the implementation of diagnostic and therapeutic approaches for sexual dysfunction as part of comprehensive patient-centered care for POTS. 

## Figures and Tables

**Table 1:** Female POTS patients’ mean scores vs. controls.

	POTS Patients *n* = 160	Controls *n* = 62	*p*	Cohen’s d
Age	30.2 ± 7.9	29.1 ± 4.6	0.5572	0.18
COMPASS-31	55.3 ± 11.8			
Orthostatic	26.2 ± 7.0			
Vasomotor	2.4 ± 1.6			
Secretomotor	9.2 ± 2.9			
GI	11.9 ± 4.4			
Bladder	2.7 ± 2.1			
Pupillomotor	2.8 ± 1.0			
Depression score	22.9 ± 10.7	8.2 ± 6.8	<0.0001	1.50
POTS-aware scoring	15.1 ± 8.6	5.9 ± 5.4	<0.0001	1.17
Loss of interest in sex	1.1 ± 1.0	0.4 ± 0.7	<0.0001	0.77
FSFI	22.4 ± 6.9	24.8 ± 5.7	0.0085	0.36
Desire	3.1 ± 1.4	4.6 ± 1.1	<0.0001	−1.18
Arousal	3.8 ± 1.5	4.9 ± 1.2	<0.0001	−0.78
Lubrication	4.0 ± 1.6	4.0 ± 1.4	0.9640	0.01
Orgasm	3.8 ± 1.6	4.2 ± 1.3	0.0986	0.23
Satisfaction	4.0 ± 1.5	4.5 ± 1.2	0.0061	−0.40
Pain	3.8 ± 1.8	2.7 ± 1.9	0.0001	0.60

**Table 2:** Male POTS patients’ mean scores compared to controls.

	POTS Patients *n* = 29	Controls *n* = 27	*p*	Cohen’s d
Age	30.1 ± 6.0	29.8 ± 5.6	0.5583	0.16
COMPASS-31	46.4 ± 11.5			
Orthostatic	21.4 ± 6.5			
Vasomotor	1.7 ± 1.7			
Secretomotor	8.8 ± 3.0			
GI	9.1 ± 4.2			
Bladder	3.0 ± 2.1			
Pupillomotor	2.5 ± 0.9			
Depression score	22.7 ± 9.8	11.0 ± 11.6	0.0002	1.09
POTS-aware scoring	16.2 ± 7.5	7.6 ± 5.9	0.0001	1.09
Loss of interest in sex	1.2 ± 1.0	0.6 ± 0.8	0.0173	0.65
IIEF	39.7 ± 11.8	54.7 ± 24.6	0.0007	−0.99
Erectile function	16.6 ± 6.0	22.0 ± 11.6	0.0011	−0.93
Orgasmic function	5.7 ± 2.7	7.6 ± 3.0	0.0170	−0.66
Sexual desire	4.4 ± 1.5	7.3 ± 2.2	<0.0001	−1.48
Intercourse satisfaction	7.1 ± 2.8	9.1 ± 4.8	0.0620	−0.52
Overall satisfaction	5.2 ± 2.0	6.9 ± 2.7	0.0118	−0.71

**Table 3:** Dysautonomia Clinic female POTS patients’ mean scores compared to controls.

	POTS Mean (SD) *n* = 11	Control Mean (SD) *n* = 7	*p*	Cohen’s d
Age	40.18 (9.37)	36.00 (13.81)	0.45	−0.37
COMPASS-31	47.55 ± 13.91			
Orthostatic	5.54 ± 2.37			
Vasomotor	3.69 ± 1.31			
Secretomotor	2.61 ± 2.50			
GI	11.15 ± 5.44			
Bladder	1.15 ± 1.57			
Pupillomotor	8.46 ± 3.78			
BDII	15.45 (10.15)	10.57 (9.54)	0.32	−0.49
FSF	19.47 (6.65)	27.08 (5.58)	0.023	1.22
Desire	2.51 (0.84)	3.34 (1.24)	0.11	0.82
Arousal	3.30 (1.59)	4.11 (0.94)	0.24	0.59
Lubrication	3.95 (1.91)	5.34 (0.96)	0.10	0.86
Orgasm	3.35 (1.86)	4.11 (1.42)	0.37	0.45
Satisfaction	3.35 (1.20)	4.74 (1.49)	0.04	1.06
Pain	3.02 (2.21)	5.43 (0.89)	0.01	1.33

**Table 4:** Pearson correlations in patients with POTS.

		COMPASS-31	BDI-II	Sexual Function Scores (FSF/IIEF)
Age	Female	−0.04, *p* = 0.5408	−0.08, *p* = 0.2700	−0.02, *p* = 0.6240
	Male	0.09, *p* = 0.6394	0.12, *p* = 0.5395	0.43, *p* = 0.0186
COMPASS-31	Female	1	0.34, *p* < 0.0001	−0.28, *p* = 0.0003
	Male	1	0.37, *p* = 0.0454	0.26, *p* = 0.1679
BDI-II	Female		1	−0.21, *p* = 0.0082
	Male		1	0.08, *p* = 0.6691
Sexual function scores	Female (FSF)			1
	Male (IIEF)			1

**Table 5:** Linear regression model for variables predicting sexual dysfunction in POTS patients.

		B	SE	95% CI	*p*
COMPASS-31	Female	−0.076	0.048	(−0.170, 0.019)	0.1155
	Male	0.254	0.193	(−0.144, 0.653)	0.2000
Age	Female	−0.058	0.067	(−0.190, 0.074)	0.3851
	Male	0.827	0.347	(0.113, 1.541)	0.0250
BDI-II	Female	−0.160	0.053	(−0.264, −0.055)	0.0030
	Male	−0.072	0.227	(−0.540, 0.397)	0.7550

## Data Availability

The original contributions presented in the study are included in the article, further inquiries can be directed to the corresponding author.
